# Known data on CoVid-19 infection linked to type-2 diabetes

**DOI:** 10.6026/97320630017772

**Published:** 2021-08-31

**Authors:** U Vidhya Rekha, M Anita, S Bhuminathan, K Sadhana

**Affiliations:** 1 Department of Public Health Dentistry, Sree Balaji Dental College and Hospital, Pallikaranai, Chennai-600100, India; 2 Department of Prosthodontics, Sree Balaji Dental College and Hospital, Pallikaranai, Chennai 600100, India

**Keywords:** CoVid-19, type-2 diabetes, Inflammatory cytokines

## Abstract

It is of interest to document the known data on CoVid-19 infection linked to type-2 diabetes. Hyperglycemia, inhibition of neutrophil chemotaxis, altered cytokine synthesis, phagocytic cell dysfunction, impaired T cell-mediated immune responses, and inadequate microbia were all seen in people with Diabetes. Individuals with diabetes have also been shown to elevate levels of the proinflammatory cytokine, especially IL-1, IL-6, and tumor necrosis factor-alpha (TNF-alpha), and different markers such as C reactive protein, D-dimer, and fibrinogen. This will prolong the cytokine storms and lead to severe illness in diabetic individuals with COVID-19 infection. The role of acute glycemic control after COVID- 19 manifestation on clinical outcomes has not been known in detail. Known data shows that hyperglycemia facilitates local viral replication in the lungs and impairs anti-viral immune response. Thus, acute glycemic management plays an important role in limiting viral replication and disease progression in patients with diabetes. The available evidence implicates diabetes as important risk factors impacting the clinical severity of SARS-CoV-2.

## Background:

COVID-19 is highly transmissible from person to person through respiratory secretions. The virus enters through mucous membranes of the upper respiratory tract, later affecting lungs [1]. In December 2019, a cluster of cases of atypical interstitial pneumonia caused by severe acute respiratory syndrome coronavirus 2 (SARS-CoV-2) was identified in Wuhan, China [2]. Corona viruses are enveloped, positive single-stranded RNA viruses widely distributed in humans and animals worldwide [3]. Although most human coronavirus infections are mild, major outbreaks of two beta corona viruses, severe acute respiratory syndrome coronavirus (SARS-CoV) in 2002-2003 and Middle East respiratory syndrome coronavirus (MERS-CoV) in 2012, have caused deadly pneumonia, with mortality rates of 10% for SARS-CoV and 36% for MERS-CoV [4]. Following the rapid spread of COVID-19, WHO on March 11, 2020, declared COVID-19, a global pandemic? Among those with severe COVID-19 and those who died, there is a high prevalence of concomitant conditions including diabetes, cardiovascular disease, hypertension, obesity, and chronic obstructive pulmonary disease [5]. Diabetes is the chronic diseases with the high prevalence globally make it a frequent comorbidity in patients with coronavirus-associated disease 2019 (COVID-19). Though diabetes increases the risk of infection in general, most studies have reported prevalence of diabetes almost similar to that in general population in patients with COVID-19. A meta-analysis of eight trials in China showed that diabetes was present in 8% of 46,248 patients with COVID-19 [6]. Diabetes with well-controlled blood glucose levels has an increased risk of infection with severe acute respiratory syndrome coronavirus 2 (SARS CoV-2) varies by region, age and ethnicity.

## CoVid-19:

The first coronavirus was discovered in 1937 in the birds and later on in the 1960s in humans [Coronavirus: Common Symptoms, Preventive Measures, and how to diagnose it [7]. The various types of viruses, capable to infect human beings are 229E, OC43, HCoV-NL63, SARS-CoV, MERS-CoV, HKU1 and SARS-CoV-2. There are several outbreaks from time to time due to these viruses. The most notorious outbreaks were in 2003, 2012, 2015 and 2018 with 774, 400, 36 and 42 deaths, respectively. It is important to mention that the 2019-2020 outbreak is started in Wuhan, Hubei Province, China in December 2019 [8] when a new strain of coronavirus was detected on 31st December 2019 [9]. World Health Organization (WHO) has given name to this virus as 2019-nCoV [10] which was later renamed as Severe Acute Respiratory Syndrome Coronavirus 2 (SARS-CoV-2) by the International Committee on Taxonomy of Viruses. The diseases caused by this virus is called as coronavirus disease 2019 and abbreviated as COVID-19 [CO: corona, VI: virus, D: disease and 19: 2019 year]. This virus was found to have 86.9% resemblance to a bat coronavirus, and, hence, is suspected to develop from bats [11]. This virus is out broken in pneumonia type of disease with respiratory problems, leading to death due to respiratory failure. About 210 countries and territories have been reported to infect with major outbreaks in the USA, China, South Korea, Italy, Iran, Japan, etc. tolling about around 163 million patients with more than 3.3 million deaths globally. The United States of America is the most affected country with the highest patients of about 32 million and about 580,468 deaths. Coronaviruses belong to the Coronaviridae family and appear just like spiked rings when observed through an electron microscope. The surface looks with various spikes, which are helpful to attack and bind living cells. These are the viruses causing the simple common cold disease to severe illnesses like Middle East Respiratory Syndrome (MERSCoV), Severe Acute Respiratory Syndrome (SARS-CoV). The source of these viruses is some animals including bats. The word coronavirus is a derivative of the Latin corona, which means crown or halo, that states to the typical look indicative of a crown or a solar corona around the virions. These viruses are having a positive-sense single-stranded RNA genome (27 to 34 kilobases) and helical symmetry nucleocapsid [12]. Typically, the coronaviruses are of ∼20 nm size draped with a large petal or club-shaped surface appearance.

Coronaviruses infect the upper gastrointestinal and respiratory tract of the mammals (including humans) and the birds. These viruses cause many diseases in animals and human beings but we are limited in this article with SARS-CoV-2, leading to COVID-19 disease. The whole clinical picture of COVID-19 is not completely known. The occurrence of the illness ranged from mild to severe. SARS-CoV-2 propagates through RNA replication using RNA-dependent RNA polymerases enzyme. This virus can mutate slowly, posing a challenge for its treatment and control. The symptoms of COVID-19 may arise within 2 to 14 days after the infection. Besides, in some cases, the diseases prevail after 27 days. However, Chinese researchers mentioned 5.2 days as an average incubation period [13].The cellular infection model is very similar to SARS-CoV. The main target of this virus is a lung and the virus spikes (binding domains) get attached to the cell receptors of the lungs. These are known as angiotensin-converting enzyme 2 (ACE2) receptors.

##  Diabetes:

Diabetic patients seem more at risk of developing severe or critical forms of COVID-19, the respective roles of diabetes per se, chronic hyperglycemia [with glycated hemoglobin (HbA1c) as proxy], insulin deficiency and/or resistance, obesity, andother comorbidities are not yet understood. Only a single study compared the clinical presentation of COVID-19 between diabetic (with or without comorbidities) and non-diabetic patients [14]. While the prevalence of diabetes among patients with COVID-19 varies from one study to another, reaching that of the general population in certain studies, there are twice as many diabetic patients among those who progress to a severe form of the infection or die from it [15]. According to Chinese data, the prevalence of diabetes in patients with a critical form of COVID-19 ranges from15 to 25%, a figure 2 to 4-fold higher than that in non-critical patients [16-18]. A prevalence exceeding 50% was even reported in the United States in patients admitted to ICU for a critical form of COVID-19 [19]. Diabetes does not seem to increase the risk of COVID-19 occurring, although diabetes is more frequent in patients with severe COVID-19. In a Chinese retrospective study, patients with diabetes had more severe pneumonia, higher concentrations of lactate dehydrogenase, α-hydroxybutyrate dehydrogenase, alanine aminotransferase, and γ-glutamyl transferase, and fewer lymphocytes with a higher neutrophil count. In the same study, a subgroup of 24 patients with diabetes had greater mortality compared to 26 patients without diabetes (16.5% vs 0%). In a prospective cohort study of patients with COVID-19 from New York City (NY, USA), the prevalence of diabetes and obesity was higher in individuals admitted to hospital than those not admitted to hospital (34.7% vs 9.7% for diabetes and 39·5% vs 30.8% for obesity, respectively) [20].

Diabetes is one of the leading causes of morbidity and mortality throughout the world. The condition is associated with several macrovascular and microvascular complications that ultimately impact the overall patient's survival. A relationship between diabetes and infection has long been clinically recognized. Infections, particularly influenza and pneumonia, are often common and more serious in older people with type 2 diabetes mellitus (T2DM). Diabetes and uncontrolled glycaemia were reported as significant predictors of severity and deaths in patients infected with different viruses, including the 2009 pandemic influenza A (H1N1), SARS-CoV and MERS-CoV. In the current SARS-CoV-2 pandemic, some studies did not find a clear association between diabetes and severe disease. However, other reports from China and Italy showed that older patients with chronic diseases, including diabetes, were at higher risk for severe COVID-19 and mortality. Scarce data exist regarding glucose metabolism and development of acute complications of diabetes (e.g., ketoacidosis) in patients with COVID-19. Infection of SARS-CoV-2 in those with diabetes possibly triggers higher stress conditions, with greater release of hyperglycemic hormones, e.g., glucocorticoids and catecholamines, leading to increased blood glucose levels and abnormal glucose variability. On the other hand, a retrospective study from Wuhan reported that around 10% of the patients with T2DM and COVID-19 suffered at least one episode of hypoglycaemia (<3.9 mmol/L). Hypoglycaemia has been shown to mobilize pro-inflammatory monocytes and increase platelet reactivity, contributing to a higher cardiovascular mortality in patients with diabetes.

## Diabetes and COVID-19:

A comprehensive report on 1099 patients in China showed a prevalence of diabetes of 7.4% in the overall COVID-19 population; however, 16.2% in those with severe disease [16]. Moreover, 26.2% of patients experiencing the primary composite end point, i.e., admission to an intensive care unit, the use of mechanical ventilation or death had diabetes, a roughly 3.6-fold enrichment in the critically affected patients. A recent meta-analysis calculated an odds ratio of 2.2 for diabetes patients to be admitted to an intensive care unit [13]. Accordingly, diabetes was significantly associated with the development of acute respiratory distress syndrome (ARDS) with a hazard ratio of 2.3 [18]. In summary, the pooled ratio of diabetes among COVID-19 patients with a more severe course compared to those with the more favourable course was 2.26 indicating a significantly elevated risk [15]. Prevalence of diabetes was about twofold increase in the non-surviving compared to the surviving COVID-19 population in China and Italy [15,5]. These data mirror the higher mortality rates of diabetes patients in SARS and MERS [21]. Moreover, presence of diabetic complications potentiates diabetes-related mortality [14]. Of note, plasma glucose levels and diabetes were independent predictors for mortality and morbidity in patients with SARS [14] but are not yet evaluated in the current COVID-19 season. In conclusion, diabetes not so much increases the risk of SARS-CoV-2 infection, but significantly enhances COVID-19 severity and mortality. The role of acute glycaemic control after COVID- 19 manifestation on clinical outcomes has not been studied yet; however, in influenza in vitro and animal data suggest that, among other negative effects, hyperglycaemia facilitates local viral replication in the lungs and impairs anti-viral immune response [22]. Therefore, acute glycemic management could play an important role in limiting viral replication and disease duration in patients with diabetes.

Diabetes patients presented with higher inflammatory serum markers including lactate dehydrogenase (LDH), creactive protein (CRP), ferritin, D-dimer, lower lymphocyte counts, and more pronounced computer tomography (CT) imaging pathologies indicating more severe overall and particularly lung involvement. The D-dimer levels, which are strongly linked to a higher mortality in COVID-19 [23], are significantly higher in patients with diabetes indicating a disposition to a hypercoagulable state [14]. One of the first reports on COVID-19 patients revealed that diabetes patients were at higher risk for need of intensive care, which usually means invasive ventilation. In this report 22.2% of intensive care unit patients had diabetes compared to 10.1% in the overall hospitalized COVID-19 population. Hence, diabetes confers a similar increase as noted for other risk populations such as those with hypertension, or cardiovascular disease [24]. Cardiac injury, defined as blood levels of cardiac biomarkers (high-sensitivity troponin I) above the 99th percentile upper reference limit, is significantly associated with mortality in COVID-19 patients. Patients with cardiac injury compared to those without had a significantly higher prevalence of diabetes (24.4% vs. 12.0%). Multivariable adjusted Cox proportional hazard regression revealed cardiac injury and ARDS, but not diabetes itself being an independent mortality risk factor. These data indicate that the adverse outcome of diabetes patients is due to a higher rate of cardiac and pulmonary complications [25]. Unfortunately, available data do not differentiate between type 1 and type-2 diabetes in COVID- 19. This makes it difficult to compare the contribution of pre-existing metabolic syndrome, as it occurs in most patients with T2DM, against hyperglycaemia without other concomitant metabolic disturbances. Retrospective data about infection rates in diabetes suggest that people with T1DMare at a greater risk for infectious disease in general, with death rates being similar to those with T2DM. Compared to matched control groups people with both diabetes types have significantly increased mortality from infectious diseases [26]. However, data stratified by type of pathogen (e.g. bacterial, viral) are currently not available.

## Conclusion:

The coronavirus disease (COVID-19) pandemic, which is caused by the extreme acute respiratory syndrome coronavirus 2 (SARSCoV-2), is causing major morbidity and mortality. Patients with diabetes mellitus, hypertension, and obesity are at a higher risk of hospitalisation and death. Several factors contribute to the high risk of COVID-19 in diabetes, including impaired immune response, hyperglycemia, inhibition of neutrophil chemotaxis, altered cytokine production, phagocytic cell dysfunction, impaired T cell-mediated immune responses, and inadequate microbial evacuation. Further research is needed to better understand the natural course of COVID-19 in diabetic patients, as well as ethnic differences in disease prevalence.
